# Artificial intelligence-enabled cardiac volumetry for opportunistic screening of cardiomegaly on chest CT: clinical validation with echocardiography

**DOI:** 10.1093/radadv/umag013

**Published:** 2026-03-07

**Authors:** Christopher M Fan, Angelo Scanio, Patricia Yokoo, Maya Wiessman, Michael Long, Matthew A Lewis, Yin Xi, Xinhui Duan, Roderick McColl, Suhny Abbara, Ronald Peshock, Fernando U Kay

**Affiliations:** Department of Radiology, University of Texas Southwestern Medical Center, Dallas, TX 75390, United States; Department of Radiology, University of Texas Southwestern Medical Center, Dallas, TX 75390, United States; Department of Radiology, University of Texas Southwestern Medical Center, Dallas, TX 75390, United States; Department of Radiology, University of Texas Southwestern Medical Center, Dallas, TX 75390, United States; Department of Radiology, University of Texas Southwestern Medical Center, Dallas, TX 75390, United States; Department of Radiology, University of Texas Southwestern Medical Center, Dallas, TX 75390, United States; Department of Radiology, University of Texas Southwestern Medical Center, Dallas, TX 75390, United States; Department of Radiology, University of Texas Southwestern Medical Center, Dallas, TX 75390, United States; Department of Radiology, University of Texas Southwestern Medical Center, Dallas, TX 75390, United States; Mayo Clinic, Cardiothoracic Imaging Division, Jacksonville, FL 32224, United States; Department of Radiology, University of Texas Southwestern Medical Center, Dallas, TX 75390, United States; Department of Thoracic Imaging, The University of Texas MD Anderson Cancer Center, Houston, TX 77030, United States

**Keywords:** artificial intelligence, cardiac volume, cardiomegaly, chest CT, echocardiography, opportunistic screening, thoracic imaging, cardiac segmentation, deep learning, incidental findings

## Abstract

**Background:**

Cardiomegaly is a clinically significant incidental finding on chest computed tomography (CT) associated with heart failure, arrhythmias, and sudden cardiac death. Qualitative radiologist assessment is variable, and automated AI tools may enable objective opportunistic cardiac volumetry.

**Purpose:**

To evaluate whether AI-enabled total cardiac volume (TCV_AI_) derived from non-ECG-gated, non-contrast chest CT can identify cardiomegaly as defined by echocardiography.

**Materials and Methods:**

This retrospective study included 307 consecutive patients (median age, 67 years; 56% male) who underwent non-contrast chest CT at a single center on 7 scanner types (4 vendors) and clinically indicated echocardiography within 31 days. A commercially available AI tool (AI-Rad Companion, Siemens Healthineers) automatically quantified TCV_AI_, indexed to body surface area (TCV_AI_/BSA). Echocardiography reports were reviewed for chamber dilation and left ventricular hypertrophy (LVH), collectively defined as cardiomegaly. Associations between TCV_AI_/BSA and echocardiographic findings were assessed using correlation, ordinal regression, and receiver operating characteristic (ROC). Interscan repeatability was evaluated in 248 patients with 544 repeat CT examinations. Prespecified sex-specific thresholds were tested in a temporally independent validation cohort of 50 patients.

**Results:**

Median TCV_AI_ was higher in patients with cardiomegaly than those without (1061.9 vs 798.4 mL; *P* < .001). TCV_AI_/BSA was associated with chamber dilation and LVH severity on univariate analysis and remained associated in multivariable ordinal models, except for right ventricular dilation. Discriminatory performance was fair to good, with area under the curve (AUC) 0.81 (95% CI, 0.75-0.87) in men and 0.77 (95% CI, 0.69-0.85) in women. Interscan repeatability was excellent (intraclass correlation coefficient [ICC]: 0.93). In independent validation, performance ranged from sensitivity 89.3%/specificity 27.3% at a high-sensitivity threshold to sensitivity 28.6%/specificity 100% at a high-specificity threshold.

**Conclusion:**

AI-derived cardiac volume from routine chest CT shows fair to good performance for identifying echocardiography-defined cardiomegaly with high measurement repeatability, supporting a potential role for automated cardiac volumetry as an objective, opportunistic biomarker.

**Abbreviations** 

AUC = area under the curve, BSA = body surface area, EMR = electronic medical record, ICC = intraclass correlation coefficient, IQR = interquartile range, LVH = left ventricular hypertrophy, NPV = negative predictive value, PPV = positive predictive value, ROC = receiver operating characteristic, TCV_AI_ = total cardiac volume, artificial intelligence derived

**Summary** 

AI-derived total cardiac volume from noncontrast non-ECG gated chest CT shows fair to good performance for identifying cardiomegaly as defined by echocardiography and enables objective opportunistic detection of cardiomegaly.

**Key Results** 

- Patients with echocardiography-defined cardiomegaly had higher AI-derived total cardiac volume than those without (median 1061.9 vs 798.4 mL; *P* < .001).
- Indexed AI-derived cardiac volume (TCV_AI_/BSA) demonstrated fair to good diagnostic performance for identifying echocardiography-defined cardiomegaly, with AUC 0.81 (95% CI, 0.75-0.87) in men and 0.77 (95% CI, 0.69-0.85) in women.
- In independent validation, performance ranged from sensitivity 89%/specificity 27% at a high-sensitivity threshold to sensitivity 29%/specificity 100% at a high-specificity threshold.

## Introduction

Cardiovascular (CV) diseases are the leading cause of death worldwide.^1^ Medical imaging offers the opportunity to identify CV diseases before clinical manifestations. The use of computed tomography (CT) is rapidly increasing globally.^2^ Chest CTs, the third most common CT, are often indicated for non-cardiac imaging; however, the cardiac image data can be used for opportunistic screening of CV diseases. Radiologists frequently encounter cardiac incidental findings because patients indicated for a chest CT often have pre-existing comorbidities such as lung disease, hypertension, and advanced age.^3^ For example, chest CTs indicated for pulmonary embolism (PE) at a single institution had incidental cardiac findings in 78.0% of scans.^4^

One such incidental cardiac finding is cardiomegaly, which has various etiologies and a high prevalence, affecting nearly 6 million Americans alone.^5^ Coronary artery disease, valvular disorders, cardiomyopathies, and congenital heart disease can cause cardiomegaly.^5^ Cardiomegaly is a predictive biomarker of morbidity and mortality for other CV diseases, such as heart failure, myocardial infarction, arrhythmias, and sudden cardiac death.^6–9^ Imaging modalities play a crucial role in diagnosing cardiomegaly, with studies demonstrating volumetric quantification of cardiac chambers using dedicated ECG-gated cardiac CT being highly accurate.^10^ These findings are supported by comparisons with established gold standards, including magnetic resonance imaging (MRI).^3^^,^^10^

For non-ECG-gated multidetector CT, cardiomegaly assessment is not yet standardized and can be imprecise, particularly when based on qualitative descriptors alone. Qualitative reporting of cardiomegaly is highly variable between radiologists and is often difficult in borderline cases.^11^ Quantitative approaches (eg, manual linear or volumetric measurements) are more objective but are often time-consuming and impractical in the workflow, especially for incidental findings.^11^ This dilemma has led to the development of automated artificial intelligence (AI) tools to rapidly estimate cardiac volume from routine chest CT, including examinations acquired on scanners from different manufacturers.^12–14^

AI has increasingly been employed to automatically extract quantitative biomarkers from chest CTs, but its accuracy and reliability are variable.^13^ In the context of cardiomegaly, data could be subject to various forms of bias due to regional variations in both the patient population and different models of CT scanners.^15^ Therefore, it is necessary to test these models across various clinical settings against established methods for assessing cardiac size.^13^ The present study aimed to evaluate whether cardiac volume measurements obtained using an FDA-cleared, commercially available AI tool on non-ECG-gated, non-contrast-enhanced chest CT could detect cardiomegaly, as defined by echocardiography, in a clinical setting of a population imaged with scanners from multiple vendors. We hypothesize a positive correlation.

## Material and methods

This retrospective analysis of prospectively collected, standard-of-care clinical and imaging data was HIPAA compliant and approved by the institutional review board (IRB), which authorized prospective data collection for retrospective evaluation of clinical efficacy AI tools in the cardiothoracic imaging domain. The cohorts analyzed in this study do not overlap with patient populations included in any previously published. The requirement for informed consent was waived. All the demographic data were extracted from the electronic medical records (EMRs).

### Cohort selection and description

The study screened 1802 consecutive patients with available medical records who underwent a non-ECG-gated non-contrast-enhanced chest CT from October 17, 2023, until December 31, 2023, in a single academic center (University of Texas Southwestern Medical Center – Dallas, TX).

The inclusion criteria for the study required participants to have undergone an echocardiogram (including transesophageal echocardiography, TEE) and a chest CT scan, performed within 31 days of each other as part of standard-of-care clinical evaluation. Because inclusion required a clinically indicated echocardiogram, the study cohort reflects a population with a higher pretest probability of CV disease. While planned exclusion criteria included missing or nondiagnostic echocardiography reports or failure of the AI tool to generate cardiac volume measurements, no patients met those criteria. Demographic variables (age, sex, race, and ethnicity) and anthropometric measurements (height, weight, and body surface area [BSA]) were extracted from the EMR closest to the echocardiography date (Epic Hyperdrive, Verona, WI, USA). The EMR was also reviewed for history of CV disease (detailed in Supplementary Methods).

### Chest CT

**Figure 1:**
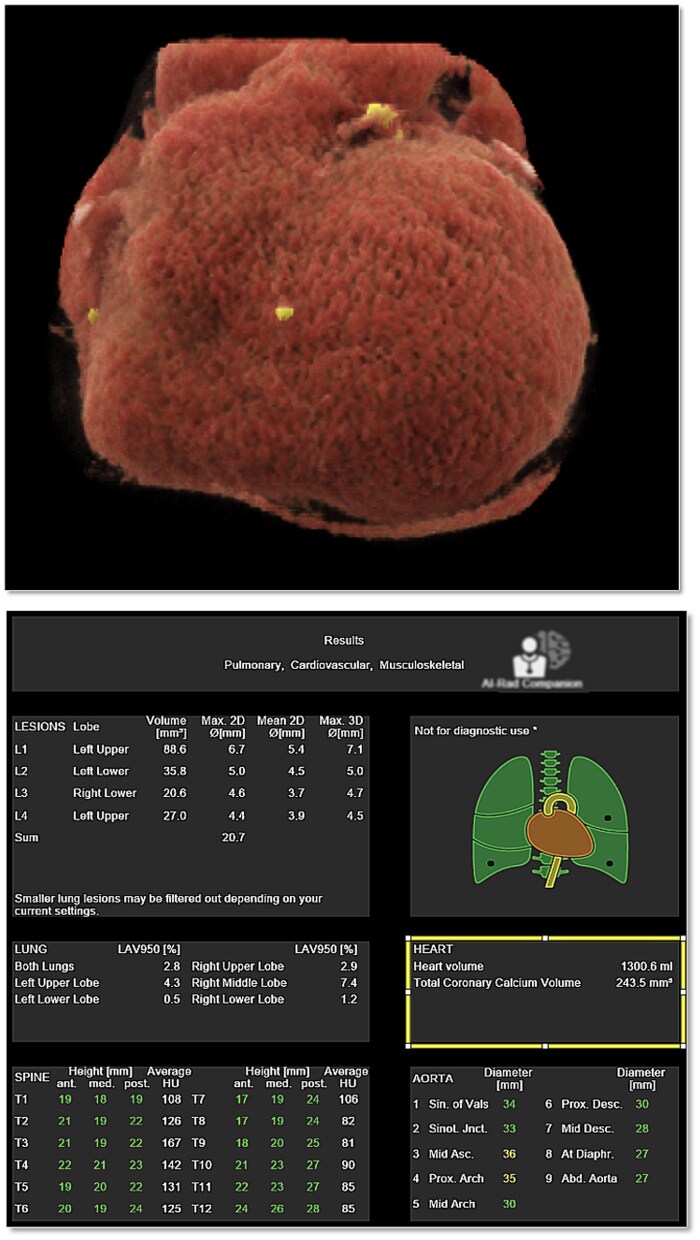
Sample output of AI-Rad Companion for non-contrast, non-ECG-gated chest CT examinations. The top image shows a 3D volume-rendered segmentation of the total cardiac volume and coronary artery calcifications. The bottom image shows the structured summary report output, with a box highlighting the cardiac findings, including “heart volume” (total cardiac volume, ie, TCVAI) and total coronary calcium volume (not analyzed in this study). In our institutional deployment, examinations are automatically routed from the CT scanners/PACS to the AI processing server as part of the standard workflow; the AI tool processes the routine soft-tissue reconstruction series used for clinical interpretation (typical reconstructed slice thickness 1-2 mm with standard kernels) and returns a structured “PDF-style” report containing embedded DICOM structured reporting elements. In this deployment, only the numeric volumetric outputs are available for downstream use; segmentation overlays are not displayed or user editable. The report can be readily extracted into the radiology reporting system to support workflow integration.

All chest CT images were obtained using standard clinical non-contrast, non-ECG-gated protocols on 7 multidetector CT scanners from 4 vendors. Detailed acquisition parameters, scanner hardware, and routing workflow are provided in the Supplementary Methods. AI-Rad Companion (FDA-cleared, Siemens Healthineers, Germany) automatically generated total cardiac volume (TCV_AI_) from non-contrast chest CT (Figure 1).^16–18^ Additional details regarding the AI model architecture, deployment configuration, and operational constraints are provided in the Supplementary Methods.

### Echocardiography

Transthoracic echocardiography (TTE) or TEE was performed by certified echocardiographers as part of routine clinical care using commercially available ultrasound systems. Studies were interpreted by board-certified cardiologists following standard institutional protocols.

Echocardiographic outcomes were abstracted from finalized clinical reports with abstraction performed in a blinded fashion with respect to CT results. Reported dilation of any of the 4 cardiac chambers, LV hypertrophy, and LVEF were recorded. Chamber size and left ventricular hypertrophy (LVH) severity were classified using an ordinal grading system based on qualitative descriptors documented in the clinical reports, with absence of dilation being 0, mild dilation being 1, moderate dilation being 2, and severe dilation being 3. Abstraction incorporated commonly used report terminology, including explicit severity labels (mild, moderate, severe) as well as widely equivalent terms (eg, *slight* or *minimal* for mild; *intermediate* for moderate; *marked*, *pronounced*, or *substantial* for severe). When dilation or hypertrophy was reported without further qualification, findings were classified as mild.

### Validation

To test the prespecified TCV_AI_/BSA thresholds in an independent dataset, we assembled a temporally distinct retrospective validation cohort consisting of the first 50 consecutive patients who underwent a non-contrast, non-ECG-gated chest CT at the same site and scanners and a clinically indicated echocardiogram performed within 1 month of each other, collected after a 3-month interval from the original cohort. All patients in the validation cohort were distinct from the derivation cohort, with no overlap between samples. For benchmarking purposes, we also abstracted from the finalized chest CT radiology reports whether cardiomegaly was documented by the interpreting radiologist.

### Interscan variability

To assess the interscan variability of TCV_AI_ measurements, we collected a separate retrospective sample of consecutive patients with at least 1 repeat non-contrast chest CT obtained between September 2022 and March 2023.

### Statistical methods

Continuous variables were reported as median and interquartile range (IQR) for non-normally distributed data. Normality was assessed using the Shapiro–Wilk test. Group differences for non-normally distributed continuous variables were evaluated using the Mann-Whitney U test. Categorical variables were summarized as counts and proportions. A *P*-value of .05 was used for significance.

Correlation and ordinal regression analyses evaluating associations between TCV_AI_/BSA and chamber-specific echocardiographic abnormalities are described in the Supplementary Methods.

Patients were later grouped by the presence of any echocardiographic abnormality. Receiver operating characteristic (ROC) analysis identified optimal TCV_AI_ cutoffs using Youden’s method. Sensitivity, specificity, positive predictive value (PPV), negative predictive value (NPV), and accuracy were calculated for various cutoffs. Areas under the curve (AUC) were estimated via bootstrapping (1000 replicates). To assess whether temporal separation between imaging studies influenced the results, sensitivity analyses excluding patients with a CT-echocardiography interval ≥7 and ≥15 days were performed.

The intraclass correlation coefficient (ICC) was calculated to assess interscan reliability for repeated measurements obtained within a 6-month interval. Reliability was interpreted as excellent (ICC > 0.9), good (0.75–0.9), moderate (0.5–0.75), or poor (< 0.5). A mixed-effects model was used to appropriately account for subjects with varying numbers of repeat measurements. Bootstrapping was applied to estimate the 95% CI around the ICC. Bland-Altman analysis was used to determine bias and 95% limits of agreement (LOAs) between repeated measurements.

Statistical analyses were performed using Python (software details are provided in the Supplementary Methods).

## Results

**Figure 2:**
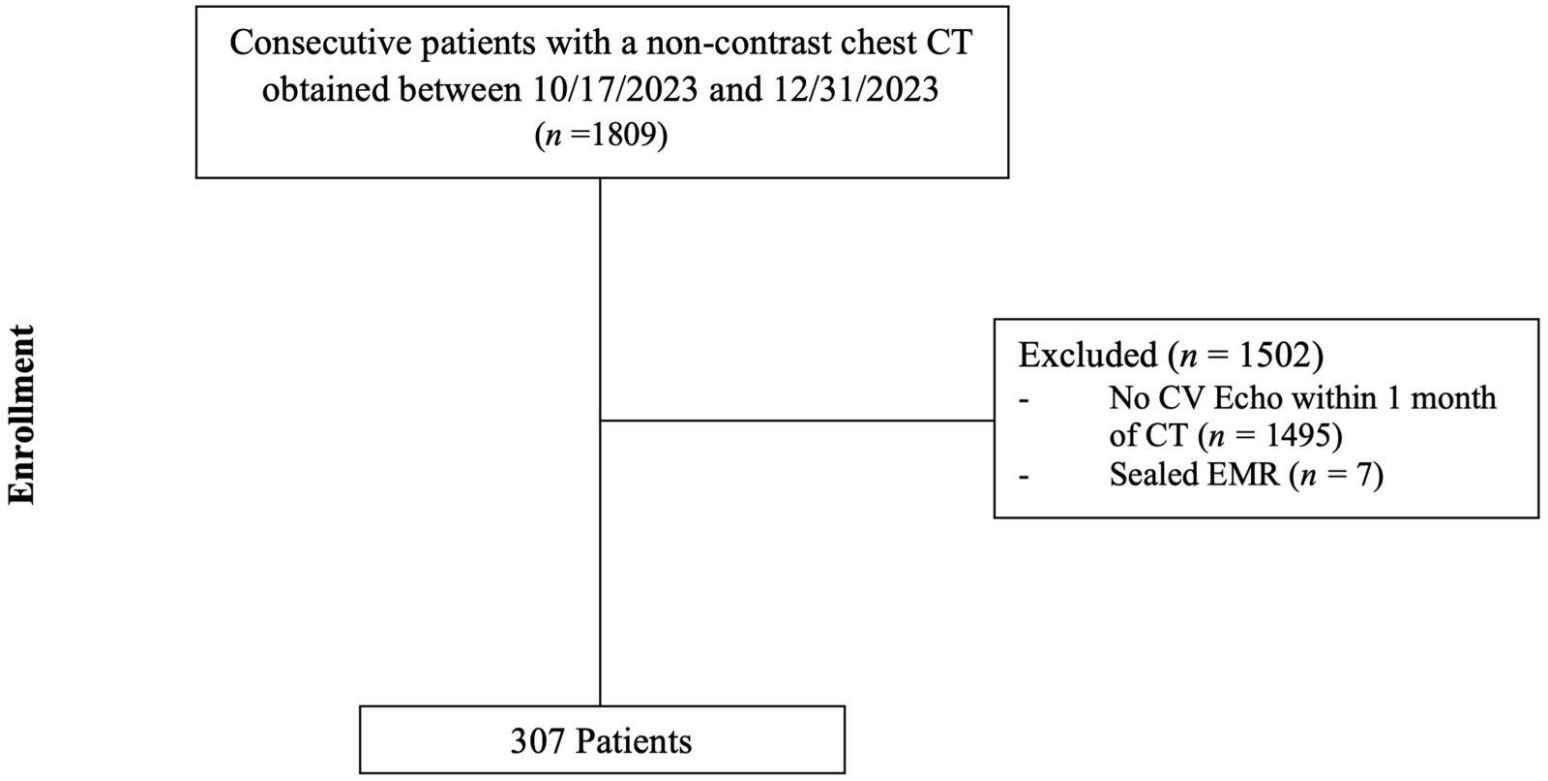
Flow diagram of patients according to eligibility and exclusion criteria. All patients undergoing a noncontrast chest CT indicated for any clinical indication according to local standard of care between October 17, 2023 and December 31, 2023 were eligible for inclusion. Patients without a cardiovascular echocardiogram obtained within 31 days of the index chest CT or with unavailable clinical or imaging information were excluded. Abbreviations: CT = computed tomography; CV = cardiovascular; EMR = electronic medical record.

A total of 307 were included as seen in Figure 2. Of the 307 patients, there were 173 males (56.3%). The median age was 67 years (IQR: 57–74, range: 25–92). The median BSA, height, and weight were 1.90 (IQR: 1.73–2.09) m^2^, 1.70 (IQR: 1.63–1.78) m, and 79.40 (IQR: 65.70–94.50) kg, respectively. Most patients had a history of CV disease (92.2%), with hypertension, arrhythmia, heart failure, and coronary artery disease being the most common conditions. The study cohort comprised the majority of White individuals, followed by Black of African American, and Asian, most of non-Hispanic or Latino ethnicity. The median time interval between echo and CT was 2 days (IQR: 1–13 days). These variables are outlined in Table 1.

**Table 1:** Patient population demographic variables.

Variable	Discovery cohort value	Validation cohort value
**N**	307	50
**Sex**	173 M (56%)/134 F (44%)	30 M (60%)/20 F (40%)
**Age (IQR)**	67 (57–74)	60 (49–68)
**History of CVD**	283 (92.2%)	42 (84.0%)
**BSA in m^2^ (range)**	1.90 (1.73–2.09)	1.92 (1.44–2.60)
**Height, in m (range)**	1.70 (1.63–1.80)	1.72 (1.51–1.91)
**Weight in kg (range)**	79.40 (65.70–94.50)	80.70 (49.0–174.20)
**Race (%)**		
**White**	213 (69.4%)	37 (74.0%)
**Black**	67 (21.8%)	8 (16.0%)
**Asian**	13 (4.2%)	0 (0.0%)
**Mixed**	1 (0.3%)	0 (0.0%)
**Unknown**	13 (4.2%)	5 (10.0%)
**Time interval between echo and CT in days (IQR)**	2 (1–13)	9 (1–15)
**CT scanner model**		
**iCT 256**	74 (24.1%)	12 (24.0%)
**SOMATOM Force**	73 (23.8%)	10 (20.0%)
**Revolution EVO**	57 (18.6%)	12 (24.0%)
**IQon—Spectral CT**	50 (16.3%)	9 (18.0%)
**Optima CT660**	22 (7.2%)	2 (4.0%)
**NAEOTOM Alpha**	19 (6.2%)	2 (4.0%)
**Aquilion**	12 (3.9%)	
**CT scanner manufacturer**		
**Philips**	124 (40.4%)	21 (42.0%)
**Siemens**	92 (30.0%)	12 (24.0%)
**GE Medical Systems**	79 (25.7%)	14 (28.0%)
**Toshiba**	12 (3.9%)	3 (6.0%)
**Chest CT—TCV_AI_ in mL (range)**	911.8 (740.0–1125.7)	923.5 (550.0–1597.5)
**LVEF (IQR)**	59% (54.5–64.0)	59% (54.0%–66.0%)

Abbreviations: BSA = body surface area; CT = computed tomography; CVD = cardiovascular disease; F = female; Hx = history; IQR = interquartile range; kg = kilogram; LVEF = left ventricular ejection fraction; m = meter; M = male; TCV_AI_ = total cardiac volume, artificial intelligence derived.

The median TCV_AI_ was 911.80 mL (IQR: 740.00–1125.70). The median TCV_AI_ was significantly higher in men (1013.10 mL) when compared to women (794.55 mL) (*P* < .001).

There were only 6 TEEs that were included. On echocardiogram, 151 patients (49.2%) had no chamber dilation or LVH. The left atrium was the most frequently enlarged cardiac chamber, observed in 99 patients (32.2%), followed by the right ventricle in 57 patients (18.5%), the right atrium in 51 patients (16.6%), and the left ventricle in 19 patients (6.2%). Table 2 summarizes the echocardiographic findings in the study cohort.

**Table 2:** Prevalence of echocardiographic abnormalities.

	*n* (total: 307)	Mild	Moderate	Severe
Right atrial dilation	51 (16.6%)	40 (58.8%)	2 (3.9%)	9 (17.6%)
Left atrial dilation	99 (32.2%)	72 (72.7%)	11 (11.1%)	16 (16.2%)
Right ventricle dilation	47 (15.3%)	39 (82.9%)	8 (17.0%)	10 (21.3%)
Left ventricle dilation	19 (6.2%)	12 (63.2%)	2 (10.5%)	5 (26.3%)
Left ventricular hypertrophy	62 (20.2%)	46 (74.2%)	10 (16.1%)	6 (9.7%)

**Figure 3:**
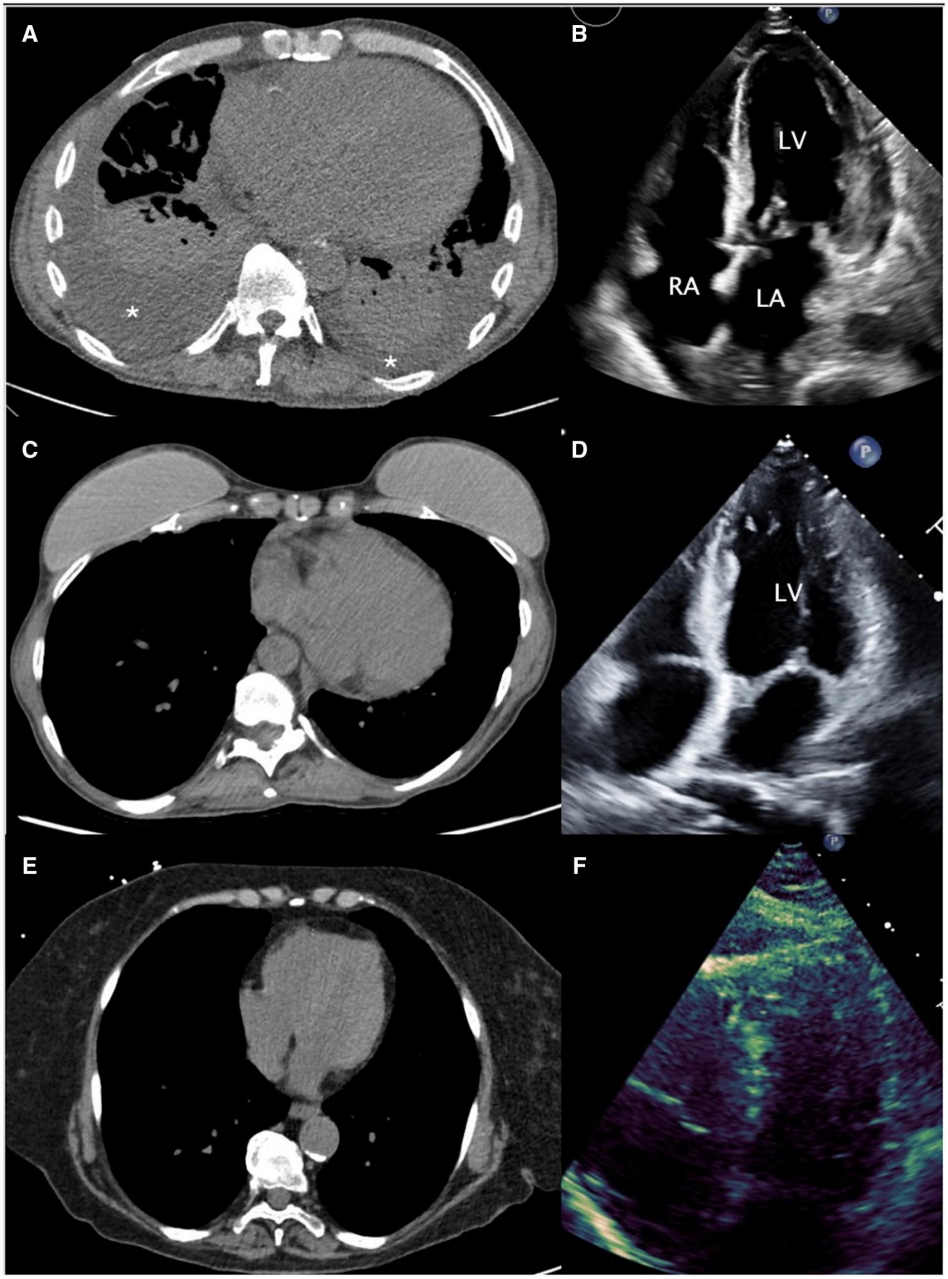
Representative examples spanning the spectrum of cardiomegaly on noncontrast chest CT with corresponding echocardiography and AI-derived total cardiac volume. (A) A 62-year-old man admitted with acute exacerbation of congestive heart failure. Chest CT demonstrates moderate right and small left pleural effusions (*) with adjacent passive atelectasis. AI-derived total cardiac volume (TCV_AI_) from the noncontrast chest CT was 1526 mL; TCV_AI_ indexed to body surface area (BSA) was 752 mL/m^2^. (B) Corresponding transthoracic echocardiography (apical 4-chamber view) demonstrates a moderately enlarged left ventricle with mild biatrial enlargement (LV = left ventricle; RA = right atrium; LA = left atrium). (C) A 62-year-old woman undergoing routine noncontrast chest CT. TCV_AI_ was 647 mL (indexed to BSA, 443 mL/m^2^). (D) Corresponding transthoracic echocardiography (apical 4-chamber view) obtained due to suspected cardiomyopathy demonstrates mild left ventricular dilation. Cardiomegaly was not reported clinically on CT; however, this case meets the high sensitivity cardiomegaly threshold (TCV_AI_/BSA ≥362 mL/m^2^). (E) A 77-year-old woman undergoing routine noncontrast chest CT to assess shortness of breath. TCV_AI_ was 461 mL (indexed to BSA). (F) Corresponding transthoracic echocardiography (apical 4-chamber view) demonstrates normal chambers.

**Figure 4:**
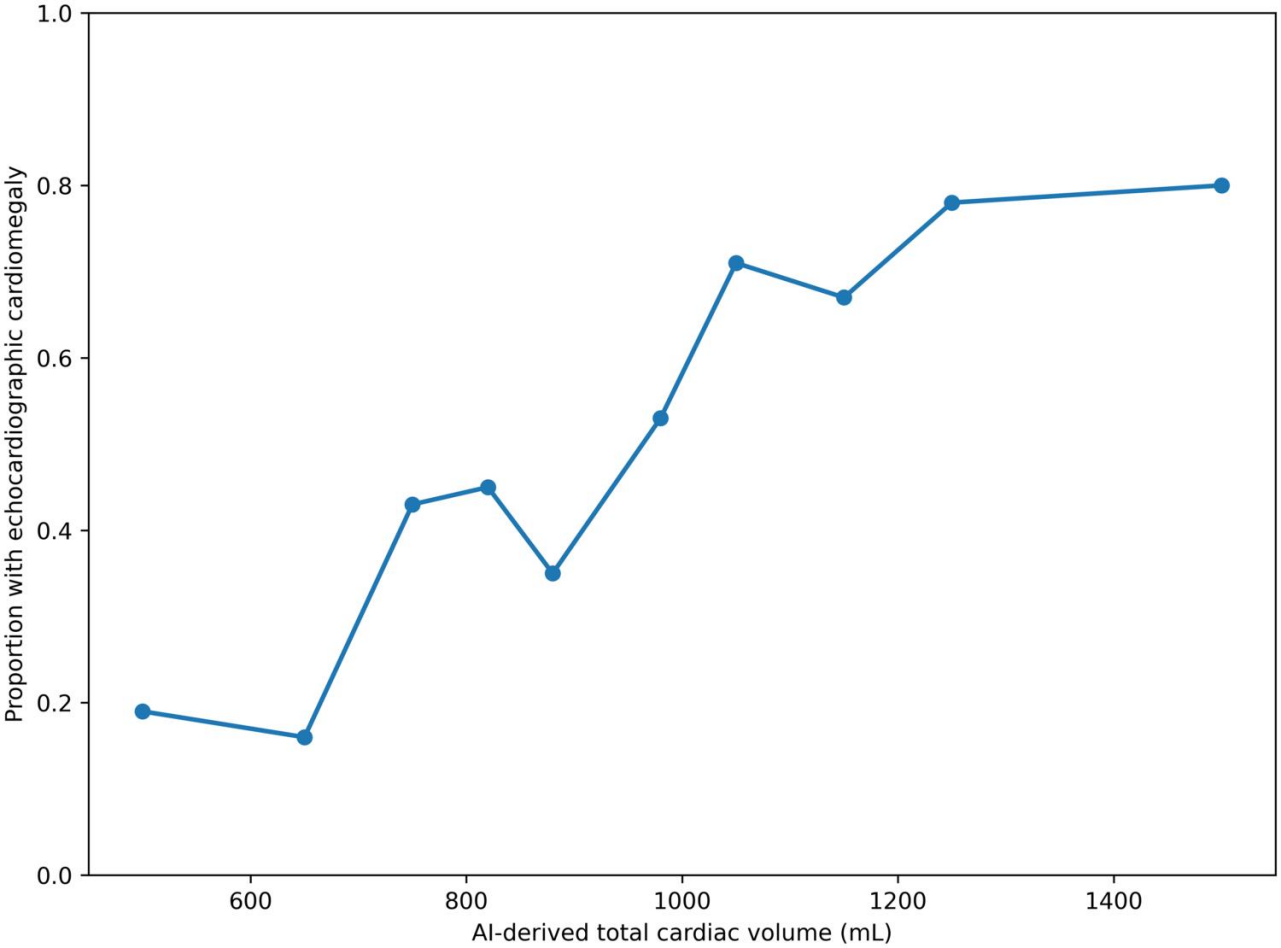
Relationship between AI-derived total cardiac volume (TCV_AI_) and echocardiographic cardiomegaly. The observed proportion of echocardiographic cardiomegaly (defined as any chamber dilation and/or left ventricular hypertrophy) is shown across deciles of TCV_AI_. Points represent the mean TCV_AI_ within each decile and the corresponding proportion of abnormal echocardiograms. The plot demonstrates a generally monotonic association without marked departures from linearity across the observed range of TCV_AI_.

**Figure 5:**
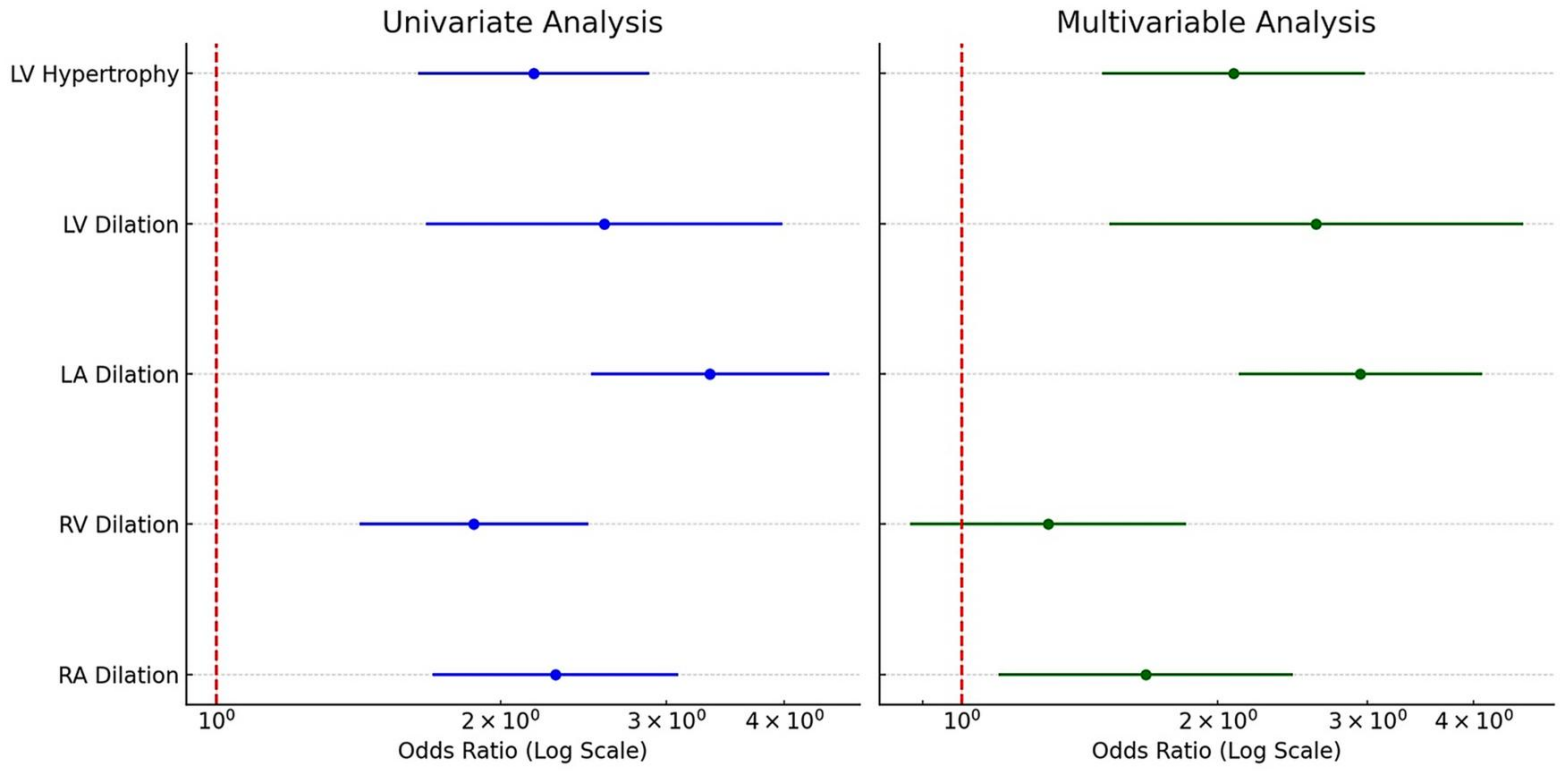
Forest plots illustrating the results of the ordinal logistic regression model using normalized TCV_AI_ (AI derived total cardiac volume)/BSA as the independent variable. The left panel presents univariate analysis, where TCV_AI_/BSA is the sole predictor of echocardiographic abnormalities (LV hypertrophy, LV dilation, LA dilation, RV dilation, and RA dilation). The right panel depicts multivariable analysis, where TCV_AI_/BSA is included alongside other echocardiographic covariates to account for concurrent abnormalities. The analysis accounts for the ordinal nature of each echocardiographic abnormality, progressing from normal to mild, moderate, and severe dilation or hypertrophy. For instance, when LV hypertrophy is the dependent variable, the model adjusts for LV dilation, LA dilation, RV dilation, and RA dilation in addition to TCV_AI_/BSA. Odds ratios represent the effect of a 1 SD increase in TCV_AI_/BSA on the likelihood of moving from a lower severity category to a higher one within the ordinal scale. Abbreviations: BSA = body surface area; LV =left ventricle; LA = left atrium; RV = right ventricle; RA = right atrium; SD = standard deviation.

TCV_AI_ in patients with normal echocardiograms was significantly lower (798.40 mL) than in patients with any echocardiographic evidence of chamber hypertrophy or dilation (1061.90 mL) (*P* < .001). Representative examples of varying severities of cardiomegaly on chest CT, along with the corresponding AI-derived cardiac volumes and echocardiographic findings, are shown in Figure 3. Visual inspection of the relationship between TCV_AI_ and echocardiographic cardiomegaly demonstrated a generally monotonic pattern without marked departures from linearity (Figure 4). To account for the association between TCV_AI_ and patient size, AI-derived values of cardiac size were indexed to BSA. The ordinal multivariable logistic regression using each isolated cardiac chamber at a time as the dependent variable revealed statistically significant associations between TCV_AI_/BSA and individual chamber enlargement or LV hypertrophy (Figure 5). Note that the statistical significance was maintained for all chamber abnormalities, except RV dilation, when the model was adjusted for the presence of concurrent abnormalities on echocardiogram.

**Figure 6:**
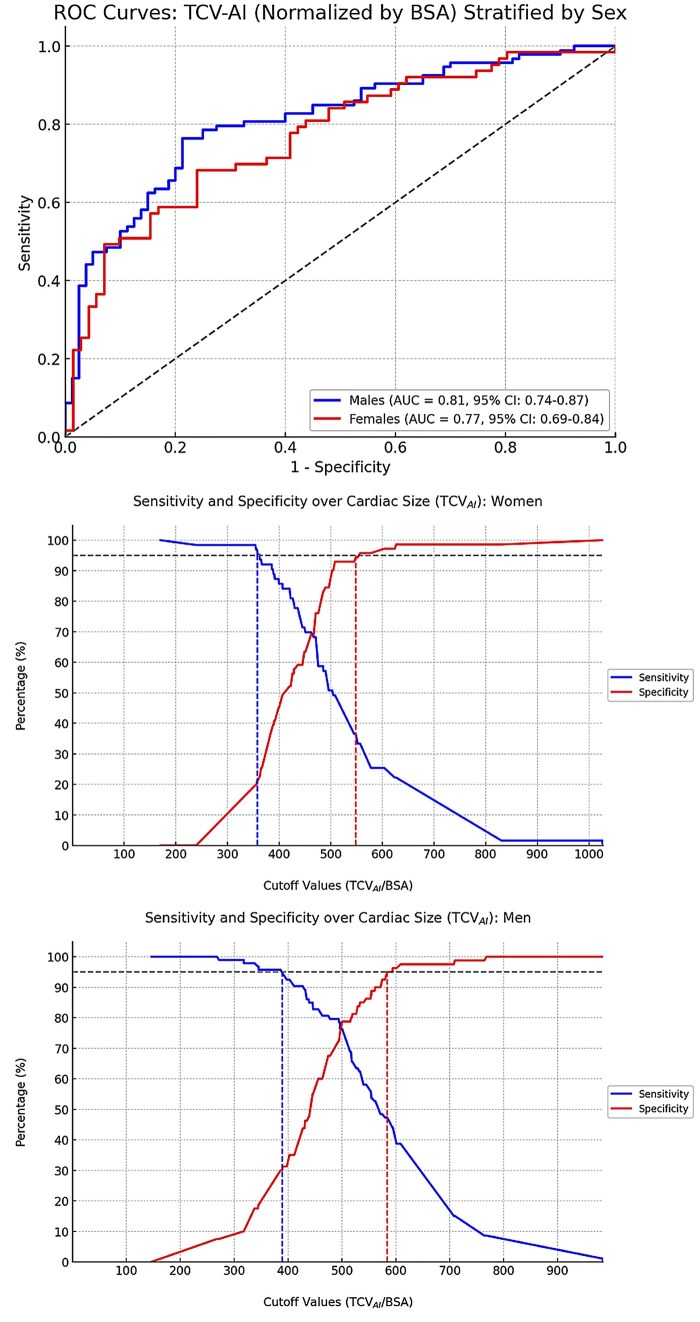
(Top) Receiving operator characteristics curve analysis for detecting any echocardiographic abnormality based on AI-derived total cardiac volume measured on chest CT (TCV_AI_) normalized to BSA. Curves were stratified by sex (males in blue and females in red). Areas under the curve with 95% CI are presented in the right lower corner. (Middle) Sensitivity and specificity curves over AI-derived total cardiac volume (TCV_AI_)/body surface area (BSA) in women. Male curves (bottom). Arbitrarily selected cutoffs of 386 mL/m^2^ for men and 362 mL/m^2^ for women would yield 95% sensitivity, but only 30.0% (95% CI, 20.3-41.3) and 22.5% (95% CI, 13.5-34.0) specificity, respectively. Cutoffs of 584 mL/m^2^ for men and 557 mL/m^2^ for women would yield 95% specificity but 46.2% (95% CI, 35.8-56.9) and 33.3% (95% CI, 22.0-46.3) sensitivity. Abbreviations: AUC = area under the curve; BSA = body surface area; ROC = receiver operating characteristic; TCV_AI_ = total cardiac volume, artificial intelligence derived.

The AUC for predicting an abnormal echocardiogram using TCV_AI_/BSA was 0.81 (95% CI: 0.75–0.87) among men and 0.77 (95% CI: 0.69–0.85) among women (Figure 6). In the ROC curve analysis, the best cutoff point was a TCV_AI_/BSA value >500 mL/m^2^ and >471 mL/m^2^ for men and women, respectively. These cutoffs, along with corresponding diagnostic performance metrics, are detailed in Table 3. Among men, this threshold yielded an accuracy of 77%, sensitivity of 76%, specificity of 79%, PPV of 81%, and NPV of 74%. Among women, the accuracy was 72%, sensitivity 68%, specificity 79%, PPV 72%, and NPV 73%. Receiver operating characteristic-derived performance based on high-sensitivity and high-specificity operating thresholds is summarized in Figure 6. Sensitivity analyses excluding patients with CT-echocardiography intervals of ≥7 and ≥15 days showed similar discriminatory performance to the primary analysis (AUC 0.79 and 0.76, respectively). Exploratory demographic-adjusted analyses are provided in the Supplementary Material.

**Table 3:** Diagnostic performance to detect any echocardiographic abnormality.

	Males	Females
AUC	0.81 [0.75–0.87]	0.77 [0.69–0.85]
Best cutoff (mL/m^2^) TCV_AI_/BSA	500	471
Sensitivity	76% [66.4–84.5] (71/93)	68% [55.3–79.4] (43/63)
Specificity	79% [68.2–87.1] (63/80)	79% [68.2%–87.1%] (63/80)

Performance metrics were derived for the best cutoff point according to Youden’s method. 95% CIs are provided within brackets. Abbreviations: AUC = area under the curve; BSA = body surface area; TCV_AI_ = total cardiac volume, artificial intelligence derived.

The prospective validation cohort included 50 consecutive patients with a median age of 60 years (range: 25–89 years). Using prespecified sex-specific TCV_AI_/BSA thresholds, diagnostic performance in the prospective cohort is summarized in Table 4. With high-sensitivity thresholds (men 386 mL/m^2^; women 362 mL/m^2^), sensitivity was 89.3%, specificity 27.3%, PPV 61.0%, NPV 66.7%, and accuracy 62.0%. With Youden-optimal thresholds (men 500 mL/m^2^; women 471 mL/m^2^), sensitivity was 60.7%, specificity 72.7%, PPV 73.9%, NPV 59.3%, and accuracy 66.0%. With high-specificity thresholds (men 584 mL/m^2^; women 557 mL/m^2^), sensitivity was 28.6%, specificity 100.0%, PPV 100.0%, NPV 52.4%, and accuracy 60.0%. For comparison, the respective radiologic reports demonstrated sensitivity 21.4%, specificity 95.5%, PPV 85.7%, NPV 48.8%, and accuracy 54.0% for the detection of cardiomegaly when evaluated against echocardiography.

**Table 4:** Diagnostic performance of prespecified sex-specific TCV_AI_/BSA thresholds in the prospective validation cohort (*n* = 50).

Operating point	Threshold (TCV_AI_/BSA, mL/m²)	Sensitivity	Specificity
High sensitivity (95%)	Men: 386/Women: 362	89.3% [71.8–97.7] (25/28)	27.3% [10.7–50.2] (6/22)
Youden-optimal (balanced)	Men: 500/Women: 471	60.7% [40.6–78.5] (17/28)	72.7% [49.8–89.3] (16/22)
High specificity (95%)	Men: 584/Women: 557	28.6% [13.2–48.7] (8/28)	100% [84.6–100.0] (22/22)

Reference standard: echocardiographic-defined cardiomegaly (any chamber dilation and/or left ventricular hypertrophy). All thresholds were derived in the original cohort and applied to a temporally distinct cohort without recalibration. 95% CIs are provided within brackets. Abbreviations: BSA = body surface area; TCV_AI_ = total cardiac volume, artificial intelligence derived.

The interscan variability sample included 248 patients (143 M/105 F, median age: 67, IQR: 60–73, range: 26–91) with 544 chest CT (details of the patient sample are provided in Table S2). The median interval between scans was 61 days (IQR: 24–91). ICC was excellent (0.93, 95% CI: 0.91–0.94); the mean bias between repeated TCV_AI_ measurements was −0.8 mL, with 95% LOA of −200 to 199 mL.

## Discussion

In this retrospective study, AI-derived heart volume measured on non-gated chest CTs performed for non-cardiac indications was associated with cardiac chamber dilation as defined by echocardiography. The TCV_AI_/BSA had fair to good diagnostic performance to identify patients with cardiomegaly, with respective AUCs of 0.81 and 0.77 in men and women. Sensitivity analyses restricting the CT-echocardiography interval to <7 days and <15 days demonstrated similar discriminatory performance, suggesting that the observed associations were not driven by temporal separation between imaging studies.

The severity of any chamber dilation or LVH was associated with TCV_AI_/BSA. When the 4 individual chamber variables and LVH were combined into a multivariable analysis, TCV_AI_/BSA was still predictive of the severity of all chamber dilations and LVH, except for right ventricular dilation. Furthermore, when separated by sex, cutoffs for TCV_AI_/BSA could be set to achieve either highly sensitive or highly specific screening for detecting possible cardiac chamber dilation or LVH, enabling tailored screening strategies based on the acceptable trade-off between false negatives and false positives.

In the multivariable analysis, the loss of RV dilation significance suggests that its predictive ability may have been overestimated in univariate models due to confounding from other chamber dilations. Several factors may explain this overestimation. Unlike the LV, there is no well-established geometric model for RV dilation on 2D echocardiography, which limits the accuracy of volumetric assessment.^19^ This has contributed to the known underestimation of RV size on echocardiography compared to cardiac MRI.^20^ Additionally, RV measurements show considerable variability between TTE and TEE due to the RV’s complex shape and orientation. Given that our study primarily relied on TTE (301 of 307 patients), this may have further contributed to the diminished correlation between TCV_AI_/BSA and RV dilation in the multivariable model.^21^

Prior work has shown a strong correlation between echocardiography-derived and CT-derived total cardiac volume, including in pediatric and young adult cohorts.^22^ In contrast, the present study evaluates opportunistic assessment in an adult population undergoing routine non-ECG-gated chest CT for non-cardiac indications, using a fully automated, FDA-cleared AI tool without manual intervention. We derived sex-specific volumetric thresholds, tested their stability in a temporally independent validation cohort, and benchmarked performance against clinically interpreted echocardiographic cardiomegaly and routine radiology reporting, aligning the evaluation with real-world workflows.

Heart chamber enlargement detection can be indicative of underlying pathologies such as heart failure, cardiomyopathy, or valvular heart disease.^6–9^ Utilizing AI based tools to assess heart size on non-gated chest CTs in a way that is seamlessly integrated into the radiologist workflow can facilitate prompt diagnosis followed by intervention without requiring extra time or resources from healthcare providers. Prior reports demonstrate how AI based heart chamber volume measurement can predict cardiac outcomes such as atrial fibrillation and stroke as well as mortality.^23^^,^^24^ Potentially, this tool will allow opportunistic identification of patients with potential cardiac abnormalities who might otherwise remain undiagnosed and can aid in risk stratification and prognostication, guiding further diagnostic and therapeutic decisions as well as management strategies.

In addition to the original analyses, we evaluated the prespecified sex-specific TCV_AI_/BSA thresholds in a temporally independent prospective cohort to assess their stability when applied to new patients imaged after completion of the derivation phase. In this cohort, the operating characteristics of the thresholds were maintained, with expected differences in sensitivity and specificity across high-sensitivity, balanced, and high-specificity operating points. These findings indicate that the associations observed in the derivation cohort were not dependent on recalibration or optimization to a single dataset and that threshold behavior remained consistent when applied to a subsequent, consecutively collected population. The attenuation in overall performance relative to the derivation cohort is consistent with application to an independent sample and reflects variability in patient mix, imaging conditions, and echocardiographic assessment in routine clinical practice.

In routine chest CT interpretation, radiologist-reported cardiomegaly tended to operate at a high-specificity/low-sensitivity point when benchmarked against echocardiography, consistent with a more conservative approach with a focus on qualitative reporting practices for incidental findings. In contrast, the availability of an automated continuous volumetric biomarker like TCV_AI_/BSA enables selection of objective prespecified operating points (eg, high sensitivity for screening/triage or high specificity for confirmatory use) based on the intended clinical task.

The following limitations need to be acknowledged. The study was retrospective in nature and performed at a single academic institution, limiting generalizability. Because inclusion required a clinically indicated echocardiogram, the derivation cohort represented a population with a higher pretest probability of CV disease rather than an asymptomatic population. Echocardiography is typically performed when there is clinical suspicion of CV pathology, which explains the high prevalence of CV comorbidities observed in this cohort. The patient cohort likely suffered from an overrepresentation of medically complex individuals. Accordingly, absolute estimates of diagnostic performance, particularly predictive values, may be influenced by disease prevalence, and direct extrapolation to lower-prevalence or screening populations should be performed with caution. Establishing normative reference ranges for cardiac volume would require prospective evaluation of asymptomatic individuals undergoing both CT and echocardiography and was beyond the scope of this clinically focused study.

Although echocardiography was used as the reference standard, chamber size assessment is limited by acoustic window dependence, complex geometry—particularly for the RV—and interobserver variability. Furthermore, reliance on report-based ordinal interpretations reflects routine clinical practice but does not provide a volumetric gold standard, which may have contributed to attenuated associations for RV dilation. In addition, only a small number of patients underwent TEE, and moderate-to-severe ventricular dilation was uncommon, which limits the precision of subgroup-specific estimates at higher severity grades; these results should therefore be interpreted with caution. Future studies should include larger multicenter comparisons across broader populations to further assess generalizability, as well as validation of additional AI tools capable of individually segmenting chamber cavities and myocardial walls on non-contrast CT scans.^25^ In our current deployment, segmentation overlays were not available for visual review or manual correction, and therefore we could not systematically adjudicate subtle segmentation failures or outliers related to image quality (eg, motion artifact).

In conclusion, our findings indicate that AI-derived CT cardiac volume is associated with clinically interpreted cardiomegaly on echocardiography. In a separate repeat-scan cohort, TCV_AI_ demonstrated excellent interscan repeatability, supporting measurement reproducibility in routine non-gated chest CT. Together, these results illustrate how automated CT-based volumetry can enable opportunistic imaging screening by providing an objective, threshold-tunable biomarker.

## Supplementary Material

umag013_Supplementary_Data

## Data Availability

The data underlying this article cannot be shared publicly for the privacy of individuals that participated in the study. Reasonable data requests can be addressed to the corresponding author.
