# Supplementary material for: Artificial intelligence-enabled cardiac volumetry for opportunistic screening of cardiomegaly on chest CT: clinical validation with echocardiography
Source: Radiol Adv. 2026 Mar 7;3(2):umag013. doi: 10.1093/radadv/umag013 (PMC13016890; doi:10.1093/radadv/umag013)
Supplement: umag013_Supplementary_Data [file umag013_supplementary_data.zip › Supplementary_Material_R2_rev_FKAY.pdf]

AI-enabled Cardiac Volumetry for Opportunistic Screening of Cardiomegaly on Chest CT: Clinical Validation with Echocardiography

Authors:

Christopher M. Fan[1], BS, christopher.fan@utsouthwestern.edu, 361-960-7690

Angelo Scanio, BS[1], angelo.scanio@utsouthwestern.edu, 832-701-5037

Patricia Yokoo, MD[1], PhD, patyokoo@gmail.com, +55(43)99928-2609,

Maya Wiessman[1], MD, maya.wiessman@utsouthwestern.edu, 214/645-7521

Michael Long[1], MS, michael.long@utsouthwestern.edu, 225-302-8168

Matthew A. Lewis[1], PhD, Matthew.Lewis@UTSouthwestern.edu, 214-648-7751

Yin Xi[1], PhD, Yin.Xi@UTSouthwestern.edu, 214-645-9004

Xinhui Duan[1], PhD, Xinhui.Duan@UTSouthwestern.edu, 214-648-3689

Roderick McColl[1], PhD, Roderick.McColl@UTSouthwestern.edu, 214-648-2910

Suhny Abbara[2], MD, abbara.suhny@mayo.edu, 214/648-0388

Ronald Peshock[1], MD, ron.peshock@utsouthwestern.edu, 214/648-7765

Fernando U. Kay[3], MD, PhD**, fkay@mdanderson.org, (Corresponding author)

Affiliations:

[1]University of Texas Southwestern Medical Center, 5323 Harry Hines Blvd, Dallas, TX 75390

[2]Mayo Clinic, 4500 San Pablo Rd S, Jacksonville, FL 32224

[3]Department of Thoracic Imaging, The University of Texas MD Anderson Cancer Center, 1515 Holcombe Blvd, Houston, TX 77030, USA

Work originated from the University of Texas Southwestern Medical Center, 5323 Harry Hines Blvd, Dallas, TX 75390

Corresponding Author:

Fernando U. Kay

The University of Texas MD Anderson Cancer Center, 1515 Holcombe Blvd, Houston, TX 77030, USA

email: fkay@mdanderson.org

Supplementary Material

Supplementary Methods

Clinical History Abstraction from Electronic Medical Records

The EMR was reviewed for history of cardiovascular disease, including aortic disease, arrhythmia, coronary artery disease, cerebrovascular disease, congenital heart disease, infective and inflammatory cardiac diseases, cardiomyopathy, cardiac valvular disease, heart failure, prior heart surgery, hypertension, pericardial disease, peripheral vascular disease, and thromboembolic disease. Clinical comorbidities were abstracted from the EMR as documented during routine clinical care and were not independently adjudicated for the purposes of this imaging-focused analysis.

CT Acquisition Hardware and Protocol Details

All chest CT examinations were performed using standard clinical non-contrast, non-ECG-gated protocols routinely employed at our institution on multidetector CT systems equipped with at least 64 detector rows. Examinations were acquired across scanners from multiple manufacturers, including GE Healthcare (Optima CT660, Revolution EVO), Philips Healthcare (iCT 256, IQon Spectral CT), Siemens Healthineers (SOMATOM Force), and Canon Medical Systems (Aquilion). Acquisition parameters varied by clinical indication (routine diagnostic chest CT, low-dose lung cancer screening, and high-resolution CT for interstitial lung disease evaluation). Scans were typically acquired at 100–120 kVp with automatic tube current modulation, and images were reconstructed using standard clinical reconstruction kernels (including soft-tissue series used for clinical interpretation and AI processing). Reconstructed slice thickness was typically 1–2 mm, depending on protocol and scanner. Images were routed from directly scanners to the AI processing server as per institutional deployment workflow.

AI Tool Description and Deployment Configuration

AI-Rad Companion (Siemens Healthineers, Germany) is an FDA-cleared, commercially available clinical software platform, compatible with multivendor chest CT data, which was used to autonomously perform segmentation of the total heart volume and generate numerical measurements in milliliters in the standard deployed version. This volume is referred to as total cardiac volume AI (TCV_{AI}). The AI, previously trained on over 650 CT datasets, uses a deep U-shaped convolutional neural network (U-Net–type) architecture [16] for automated cardiac segmentation and reports total cardiac volume [17], as illustrated in Figure 2. Prior clinical evaluations of this AI platform applied to noncontrast chest CT imaging have been reported elsewhere [18]. The present study was designed to evaluate the clinical associations and threshold-based performance of the resulting volumetric output, rather than to revalidate algorithm development or internal training/validation procedures of this FDA-cleared tool. In our institutional deployment, only the numeric cardiac volume output is returned, and segmentation overlays are not displayed or user-editable; consequently, systematic visual review of segmentations to identify failures modes was not feasible, and analyses were limited to cases in which a numeric output was generated. The evaluated system performs fully automated cardiac segmentation and volumetric quantification without user-adjustable contours or manual post-processing. Cardiomegaly was not classified directly by the software; instead, cardiomegaly screening was inferred by applying prespecified, statistically derived thresholds to total cardiac volume indexed to body surface area, as described below.

Left Ventricular Ejection Fraction Analysis

The LVEF was documented for available patients. The average (rounded to the nearest percent) was recorded if given a range. If the LVEF was described as "severely depressed," 34.0% was noted, as it is less than 35.0%. If a percentage was documented with a “>” or “<” symbol, the nearest whole number was recorded (e.g., for ">70.0%", 71.0% was recorded).

Correlation Analyses

Spearman's rank correlation (ρ) assessed associations between non-normally distributed continuous variables, with strength interpreted as very weak (ρ = 0.00–0.19), weak (0.20–0.39), moderate (0.40–0.59), strong (0.60–0.79), and very strong to perfect (0.80–1.00).

Original Regression Modeling

Ordinal logistic regression was used to evaluate the association between indexed cardiac volume (TCV_{AI}/BSA) and the severity of echocardiographic abnormalities, modeled on an ordinal scale (none, mild, moderate, severe). Separate models were fit for each echocardiographic outcome (left ventricular hypertrophy, left ventricular dilation, left atrial dilation, right ventricular dilation, and right atrial dilation). Univariate models included TCV_{AI}/BSA as the sole predictor. In the corresponding multivariable models, TCV_{AI}/BSA was included together with the other echocardiographic chamber abnormalities (i.e., the remaining four outcomes) as covariates to account for the frequent coexistence of structural abnormalities across chambers. This approach was used to assess whether TCV_{AI}/BSA remained associated with the severity of a given chamber abnormality independent of concurrent abnormalities in other chambers.

Sensitivity Analyses for CT–echocardiography Interval

Because echocardiography is clinically indicated and may be performed days to weeks after chest CT, sensitivity analyses were conducted to evaluate whether temporal separation between imaging studies influenced the observed association between TCV_{AI}/BSA and echocardiographic cardiomegaly. Two restricted cohorts were evaluated by excluding patients with CT–echocardiography intervals of ≥7 days and ≥15 days, respectively, thereby assessing the stability of results under progressively stricter temporal alignment.

Exploratory Multivariable Logistic Regression Including Demographic Covariates

To address the incremental contribution of AI-derived cardiac volume beyond basic demographic information, we performed an exploratory multivariable logistic regression analysis. The binary outcome was echocardiographic cardiomegaly, defined as the presence of any chamber dilation and/or left ventricular hypertrophy.

A base model was constructed including age (continuous), sex (binary), and ethnicity (categorical, one-hot encoded with reference category). A full model additionally included AI-derived total cardiac volume indexed to body surface area (TCV_{AI}/BSA, continuous). Body surface area was not included as a separate covariate because the primary predictor was already indexed.

Model discrimination was assessed using the AUC. The incremental change in AUC (ΔAUC) between the base and full models was estimated, and uncertainty was quantified using bootstrap resampling (1,000 iterations). This analysis was exploratory and intended to contextualize the contribution of TCV_{AI}/BSA rather than to develop a clinical risk prediction model.

Statistical Software and Packages

Analyses were conducted using Python (version 3.11.8) within a Jupyter Notebook environment, utilizing the Pandas (1.5.3), Matplotlib (3.6.3), Seaborn (0.11.2), SciPy (1.15.2), and NumPy (2.2.4) libraries.

Supplementary Results

Correlation Analyses

TCV_{AI} was weakly to moderately correlated with height (p = .38, 95% CI: 0.27–0.47) and moderately correlated with weight (p = .52, 95% CI: 0.43–0.61) and BSA (p = .53, 95% CI: 0.43–0.61) (Supplementary Figure S1). Correlations among chamber-specific echocardiographic abnormalities, left ventricular hypertrophy, and TCV_{AI} are summarized in Supplementary Table S1. The overall patient

population had a median LVEF of 59.0% (IQR: 54.5-65.0%). When comparing TCV_{AI}/BSA to LVEF there was a very weak inverse correlation (ρ: -0.16, 95% CI: -0.27, -0.04, p = .005) seen in Supplementary Figure S2.

Sensitivity Analyses

After exclusion of patients with a CT–echocardiography interval of ≥7 days, the restricted cohort included 192 patients. In this cohort, the association between TCVAI/BSA and echocardiographic cardiomegaly was preserved, with discriminatory performance comparable to that of the full cohort (AUC 0.79 vs 0.79 in the primary analysis).

After exclusion of patients with a CT–echocardiography interval of ≥15 days, the restricted cohort included 239 patients. In this cohort, discriminatory performance remained similar, with only modest attenuation (AUC 0.76).

Collectively, these findings indicate that the primary results were not driven by longer CT–echocardiography intervals or potential interval clinical change.

Incremental Discrimination with Addition of TCV_{AI}/BSA

The base model including age, sex, and ethnicity demonstrated limited discrimination for echocardiographic cardiomegaly (AUC = 0.63). Addition of TCV_{AI}/BSA to the model resulted in a substantial improvement in discrimination, with a full-model AUC of 0.79.

The mean ΔAUC was 0.16, with a bootstrap 95% confidence interval of 0.10–0.23, indicating a meaningful incremental contribution of AI-derived cardiac volume beyond demographic variables alone. These findings suggest that TCV_{AI}/BSA provides information not captured by age, sex, and ethnicity in this cohort.

Supplementary Discussion

Weak Correlation between TCV_{AI}/BSA and Left Ventricular Ejection Fraction

We also observed a very weak inverse correlation between TCV_{AI}/BSA and LVEF, which is clinically expected because global cardiac size and systolic function reflect related but distinct phenotypes, and cardiomegaly may be present despite preserved ejection fraction.

Pathways for Clinical Use of TCV_{AI}

Patients undergoing chest CT for non-cardiac indications may benefit from added opportunistic screening for cardiac enlargement. By selecting a lower TCV_{AI}/BSA threshold, sensitivity can be increased at the cost of specificity — a trade-off common in other screening paradigms. For instance, in low-dose CT-screening for lung cancer, Lung-RADS v1.1, achieves high sensitivity (89–93%) for intermediate-sized nodules (6–10 mm), while accepting lower specificity (26–31%) to minimize missed cancers [1]. Even with improvements in volumetric criteria, and expanding the definition of benign morphologic features, the specificity improved to 47–59% with minimal impact on sensitivity [1]. This reflects a core screening principle: thresholds prioritize early detection, tolerating more false positives to maximize benefit. Similarly, our automated cardiac volume analysis emphasizes sensitivity to flag cardiomegaly or chamber enlargement, with potential refinement through sex-specific cutoffs and downstream diagnostic confirmation.

Supplementary References

1. Hammer MM, Hunsaker AR. Strategies for Reducing False-Positive Screening Results for Intermediate-Size Nodules Evaluated Using Lung-RADS: A Secondary Analysis of National Lung Screening Trial Data. AJR Am J Roentgenol. 2022 Sep;219(3):397–405.

Supplementary Tables

Supplementary Table S1. Spearman Correlation Matrix

	TCV_{AI}	RA dilation	LA dilation	RV dilation	LV dilation	LV hypertrophy
**TCV_{AI}**	1.000	0.221	0.402	0.244	0.241	0.252
**RA dilation**	0.221	1.000	0.329	0.562	0.163	0.033
**LA dilation**	0.402	0.329	1.000	0.220	0.199	0.234
**RV dilation**	0.244	0.562	0.220	1.000	0.113	0.104
**LV dilation**	0.241	0.163	0.199	0.113	1.000	0.264
**LV hypertrophy**	0.252	0.033	0.234	0.104	0.264	1.000

Note: Spearman rank correlation coefficients (ρ) describing associations between echocardiographic chamber dilation severity grades (0–3), left ventricular hypertrophy, and AI-derived total cardiac volume (TCV_{AI}).

Supplementary Table S2. Interscan Variability Cohort Characteristics and CT Equipment

Category	Variable	Value
**Cohort characteristics**	Number of patients	248
	Sex	143 male (57.7%)<br>105 female (42.3%)
	Age, years	67 (IQR 60–73)
	Number of chest CT studies	544
	Interval between scans, days	61 (IQR 24–91)
	TCV_{AI} at baseline CT	894.4 (IQR 734.7–1068.1)
**CT Scanner Model**	Optima CT660	138 (25.4%)
	iCT 256	115 (21.1%)
	Revolution EVO	111 (20.4%)
	IQon – Spectral CT	83 (15.3%)
	SOMATOM Force	67 (12.3%)
	Aquilion	28 (5.1%)
	NAEOTOM Alpha	1 (0.2%)
	Ingenuity CT	1 (0.2%)
**CT Manufacturer**	GE Medical Systems	249 (45.8%)
	Philips	199 (36.6%)
	Siemens	68 (12.5%)
	Toshiba	28 (5.1%)

Note: Values are reported as n (%) or as median (IQR, interquartile range). TCV_{AI} indicates artificial intelligence–derived total cardiac volume in mL.

Supplementary Figures

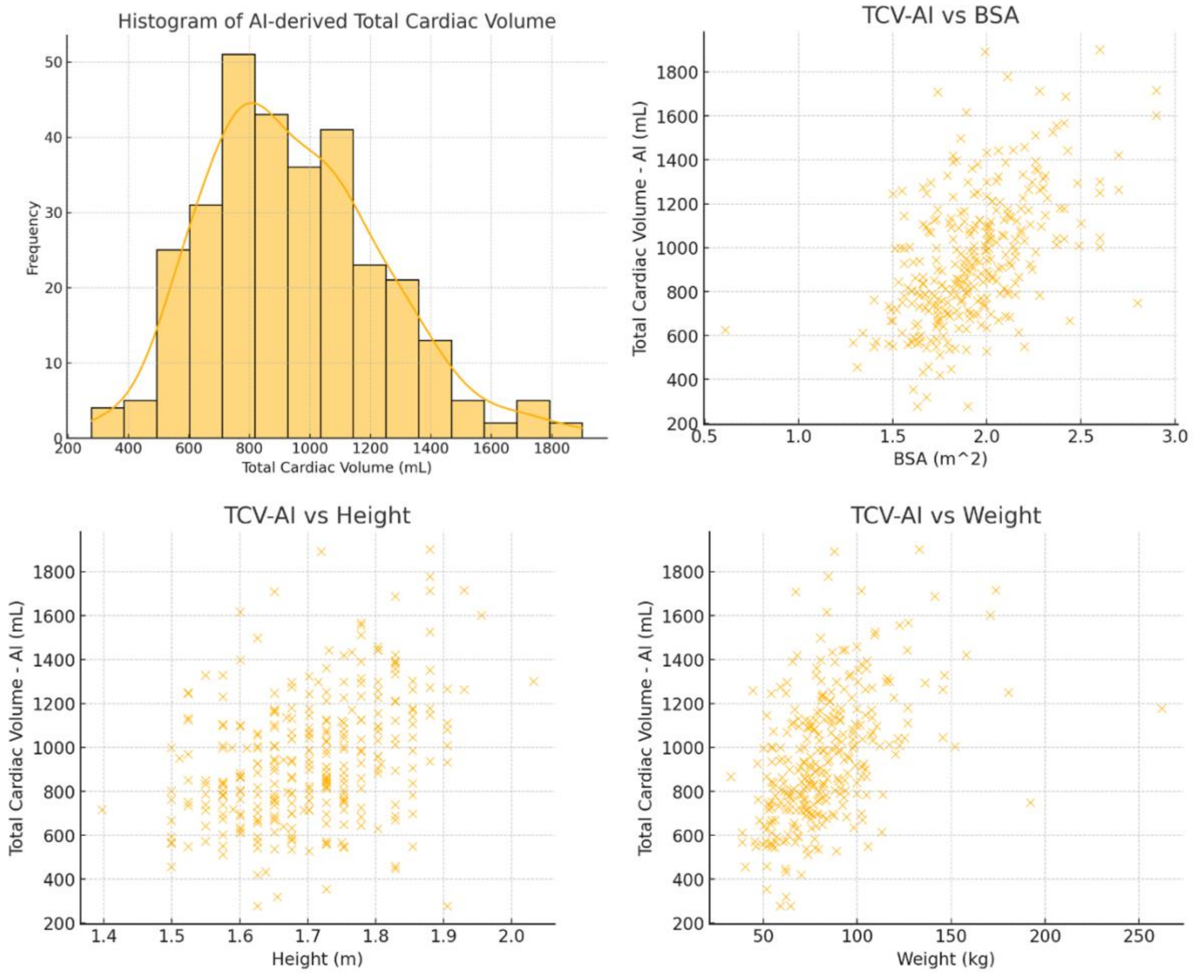

Figure S1: Histogram showing the distribution of total cardiac volume (TCV_{AI}) in the study cohort (a). Scatter plots between TCV_{AI} and BSA(b), height (c), and weight (d).

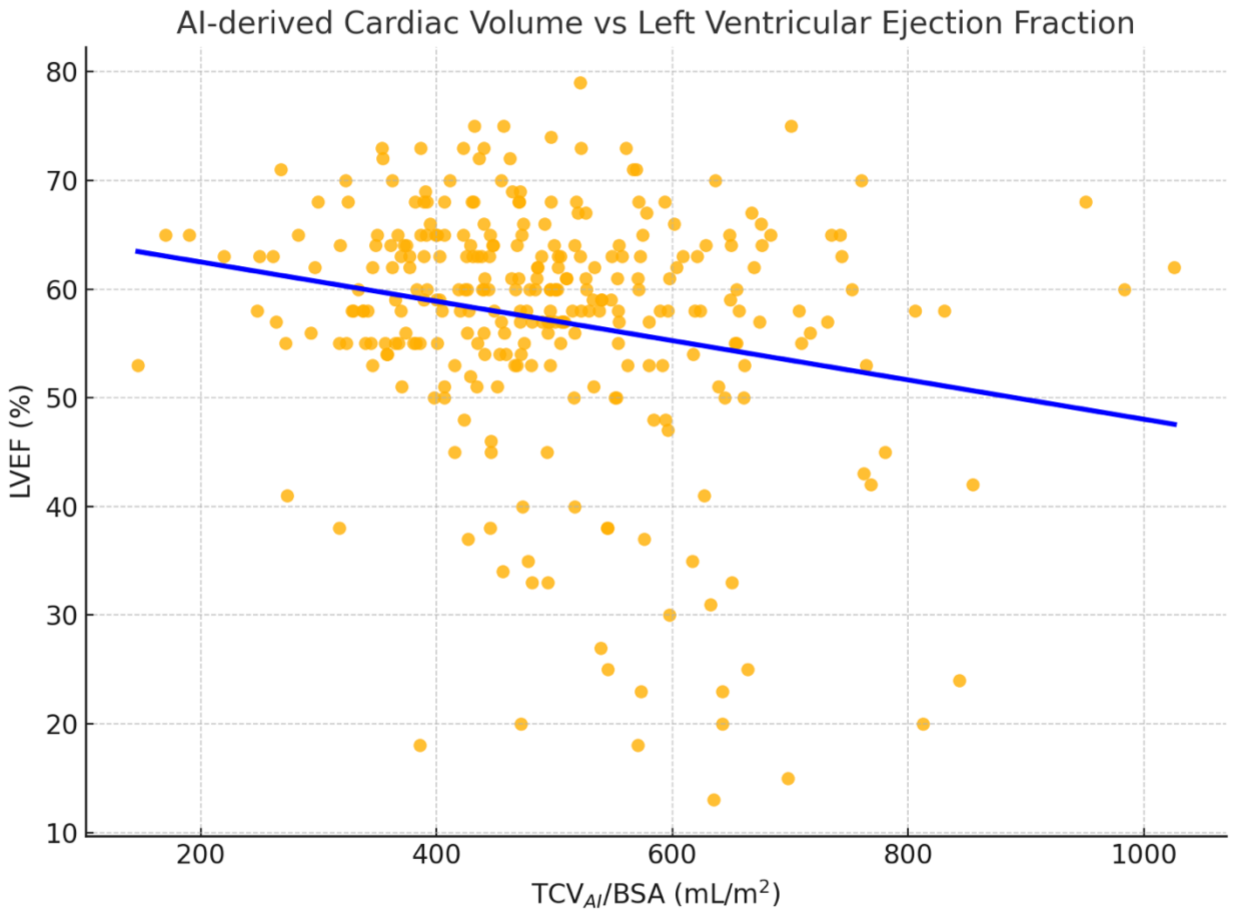

Figure S2: Scatterplot showing the negative correlation of AI-derived total cardiac volume/body surface area (TCV_{AI}/BSA) with left ventricular ejection fraction (LVEF), with a Spearman rank correlation coefficient of -0.16 (95% CI: -0.27, -0.04, $p = .005$).
